# Transcriptome analysis of the aged SAMP8 mouse model of Alzheimer’s disease reveals novel molecular targets of formononetin protection

**DOI:** 10.3389/fphar.2024.1440515

**Published:** 2024-08-21

**Authors:** Bo Liu, Di Cui, Jie Liu, Jing-Shan Shi

**Affiliations:** Key Lab for Basic Pharmacology and Joint International Laboratory of Ethnomedicine of Ministry of Education, Zunyi Medical University, Zunyi, China

**Keywords:** RNA-seq analysis, aged SAMP8 mouse, Alzheimer’s disease, formononetin, ingenuity pathway analysis

## Abstract

**Background:**

Senescence-accelerated mouse prone 8 (SAMP8) and age-matched SAMR1 mice are used to study the pathogenesis and therapeutics of Alzheimer’s disease (AD); however, the molecular mechanisms are not completely understood.

**Objective:**

This study aimed to examine the effects of the 5-month administration of formononetin in SAMP8 mice and used RNA-seq to explore the molecular targets.

**Methods:**

SAMP8 mice were orally administered formononetin (0, 8, and 16 mg/kg) from 4 months of age, and age-matched SAMR1 mice were used as controls. Behavioral tests were performed in 9-month-old mice, followed by histopathologic analysis. Total RNA from the hippocampus was isolated and subjected to RNA-seq, RT-qPCR, and bioinformatics analysis.

**Results:**

The 9-month-old SAMP8 mice exhibited cognition deficits, evidenced by novel object recognition, open-field test, elevated plus maze, and passive avoidance. Nissl bodies in the cortex and hippocampus were decreased. Formononetin treatments ameliorated behavioral deficits and improved morphological changes, which were evidenced by Nissl and H&E staining. RNA-seq revealed distinct gene expression patterns between SAMP8 and SAMR1 mice. Differentially expressed genes in SAMP8 mice were attenuated or normalized by formononetin. Ingenuity pathway analysis (IPA) of canonical pathway and upstream regulators revealed increases in proinflammatory factors and immune dysfunction and decreases in NRF2 and SIRT-1 signaling pathways, leading to neuroinflammation. Formononetin treatment attenuated or reversed these molecular changes. The transcriptome of SAMP8 mice was correlated with transcriptomic profiles of other AD mouse models in the GEO database.

**Conclusion:**

Neuroinflammation and decreased antioxidant and SIRT-1 signaling contributed to cognitive deficits in aged SAMP8 mice, which are potential therapeutic targets of formononetin in combination with other therapies.

## 1 Introduction

Alzheimer’s disease (AD) is common in the elderly, and the main clinical manifestation is the decline in learning and memory functions (Hu et al., 2023). It is also characterized by neuropsychiatric symptoms including psychological distress and alterations in mood, including anxiety and depression (Botto et al., 2022). Senescence-accelerated mouse prone 8 (SAMP8) is a useful model to study learning dysfunction and anxiety deficiency, showing pathological changes and clinical symptoms similar to those of AD (Pačesová et al., 2022). Therefore, SAMP8 mice and age-matched SAMR1 mice have been widely used to enhance our understanding of AD and provide an effective way to find new therapeutic targets. We have reviewed the SAMP8 mouse model to study AD mechanisms (Liu et al., 2020b) and used this model to screen potential therapeutics from active ingredients of Chinese medicines such as icariin from *Epimedium* (Chen et al., 2019) and DNLA from *Dendrobium nobile* Lindl. (Liu et al., 2020a; Lv et al., 2020).

Formononetin (FMN) is a naturally occurring isoflavone phytoestrogen, which is the bioactive constituent of numerous medicinal plants such as licorice, Radix puerariae, *Trifolium pratense*, *Astragalus membranaceus*, *Angelica sinensis*, Kudzu root, and *Caulis spatholobi* (Tian et al., 2022; Singh et al., 2023). FMN has a similar structure to that of β-estradiol, and emerging evidence indicates that FMN possesses considerable anti-inflammatory, antioxidant, anti-tumor, neuroprotective, and estrogen-like effects (Aboushanab et al., 2022). The neuroprotective properties of FMN have been observed in multiple neurological disorders, including traumatic brain injury, ischemic stroke, AD, dementia, anxiety, and depression (Ma and Wang, 2022; Tian et al., 2022; Singh et al., 2023). FMN significantly improved learning and memory functions by suppressing Aβ production from APP processing in APP/PS1 AD mice (Fei et al., 2018). Our recent studies have also demonstrated that FMN delayed senescence in *C. elegans* (Cui and Shi, 2023). However, the effects and mechanisms of FMN on the SAMP8 AD mouse model have not been studied.

High-throughput technology greatly facilitated our understanding of AD (Aerqin et al., 2022). We have used proteomics to analyze the protective effects and mechanisms of DNLA in aged SAMP8 mice (Liu et al., 2023). RNA-seq and miRNA-seq are used to study the UNC0642 inhibition of the G9a/GLP complex in SAMP8 mice (Pawar et al., 2021). We have used RNA-seq technology to analyze the molecular mechanisms by which FMN protects against cognitive deficits in aged SAMP8 mice. First, the effects of FMN on cognitive function, psychology, and mood of SAMP8 mice were observed, and second, Nissl and H&E staining were used to evaluate the neuronal damage and neuron loss. Finally, total RNA from the hippocampus was extracted for RNA-seq. Comprehensive bioinformatics including Partek Flow, ingenuity pathway analysis, and Illumina BaseSpace Correlation Engine were used to determine the biological significance of differentially expressed genes, and selected DEGs were verified via RT-qPCR. Several molecular events such as neuroinflammation and decreased antioxidant and SIRT-1 signaling could contribute to cognitive deficits in aged SAMP8 mice and are potential targets of therapeutics like FMN.

## 2 Materials and methods

### 2.1 Animals

Four-month-old male SAMP8 and SAMR1 mice were purchased from Peking University (SCXK 2016-0010) and fed in a specific pathogen-free (SPF) animal room (SYXK 2014-003) at the Key Laboratory of Basic Pharmacology of Zunyi Medical University. In order to rule out the effect of sex on the molecular mechanisms of mice, we used male mice, as in our prior publications (Lv et al., 2020; Liu et al., 2023). During the experiment, a controlled 12-h light/dark cycle, room temperature at 22°C–25°C, and relative humidity of 50%–70% were maintained. Drinking water and rodent food were provided *ad libitum*. The animal experimental procedures complied with the Chinese Animal Care and Use Guidelines, and the research protocol has been approved by the Institutional Animal Use and Care Committee (Ethics No. [2020]2-142).

### 2.2 Drugs

Formononetin (FMN, C₁₆H₁₂O₄; molecular weight: 268.26; purity ≥98%) was purchased from Shanghai Jizhi Biochemical Technology Co., Ltd. (Shanghai, China). FMN was dissolved in normal saline (NS) and diluted to the concentrations of 8 mg/kg and 16 mg/kg. All reagents were of analytical reagent grade and were commercially available.

### 2.3 Experimental design

Mice were randomly divided into SAMR1, SAMP8, SAMP8 + FMN-L (8 mg/kg), and SAMP8 + FMN-H (16 mg/kg) groups, with 10 mice in each group. The dose selection of FMN was done based on previous publications (Tian et al., 2022; Pingale and Gupta, 2023). The SAMR1 and SAMP8 groups were given FMN by gavage for 5 months, starting at 4 months of age. The SAMP8 + FMN-L and SAMP8 + FMN-H groups received normal saline (NS). The mice were given oral FMN once a day. Behavioral experiments were performed at 9 months of age (Pačesová et al., 2022). After behavioral tests, the mice were euthanized, the brains of three mice in each group were fixed for pathological examination, and the hippocampi of the remaining brains were frozen at −80°C for RNA-seq and RT-qPCR analysis.

### 2.4 Behavioral tests

#### 2.4.1 Novel object recognition test

The novel object recognition (NOR) test was used to test the learning and memory abilities. The NOR test is composed of a reaction box (length 40 cm, width 40 cm, and height 45 cm), camera equipment, and behavioral analysis software (TopScan, Clever Sys Inc., Reston, VA, United States). On the first day, each mouse was placed on the reaction box for 10 min to be familiar with the environment. On the second day, two yellow cylinders with a diameter of 3 cm (A + A) were located at the two corners of the reaction box, and rodents were released against the center of the opposite wall with its back to the cylinders and were allowed to freely explore the reaction box for 5 min. At the same time on the third day, one cylinder was replaced by a novel blue cone (B). All of the mice were allowed to freely explore the reaction box for 5 min. The distance between the mouse’s nose and the novel blue cone within 2 cm was defined as exploratory behavior. The novel blue cone exploration time was recorded (Nogueira et al., 2024).

#### 2.4.2 Open-field test

The locomotor activity and anxiety-like behavior were evaluated by the open-field test (OFT). A cuboid box (25 cm length × 25 cm width × 35 cm height) was used to perform the OFT. The bottom of the box was mapped by the system and divided into the center and periphery. Before the test, the mice were habituated to the experimental environment for 30 min, and they were allowed to familiarize themselves with the apparatus for 10 min. Afterward, the mice was placed in the center, and The Top View Animal Behavior Analyzing System (Version 3.00, Clever Sys Inc., United States) was used to record the movement in 10 min. The cuboid box was cleaned with 70% alcohol before the test of another animal. The time spent in the center area was calculated (Yang et al., 2024).

#### 2.4.3 The elevated plus maze test

The elevated plus maze (EPM) test was used to elevate the anxiety disorders. The EPM apparatus consisted of two open arms and two closed arms (35 cm length × 5 cm width × 15 cm height), and the maze length was about 50 cm. Mice were put in the central area, with the head toward the open arm, and allowed to explore freely for 10 min. The Top View Animal Behavior Analyzing System (Version 3.00, Clever Sys Inc., United States) was used to record the time spent in open arms. The arms were cleaned with 70% alcohol before the testing of another animal. The time spent in the open arms was calculated (Su et al., 2024).

#### 2.4.4 Passive avoidance test

The passive avoidance test (PAT) is a fear-motivated test, which is classically used to assess memory. The PAT apparatus is composed of a light box and a dark box. The mice were put into the light box and were allowed to explore freely for 10 s to familiarize themselves with the environment. In addition, the door between the light box and the dark box was opened, and the mice would explore the dark box through the door because of their dark-loving nature. After entering the dark box, the mice were given electricity treatment to induce painful memories of electrical stimulation (shock time 2 s and current 0.2 mA). After 24 h of training, the current in the dark box was turned off, and the mice were put back into the PAT apparatus. The time spent by the mice in the light box within 5 min was recorded (Stepnik et al., 2024).

### 2.5 Hematoxylin–eosin staining

After behavioral testing, the mice were anesthetized, and brains were perfused transcardially with ice-cold NS and 4% paraformaldehyde. After that, the brain tissue was removed and soaked in 4% paraformaldehyde at 4°C for 24 h. The coronal sections of the hippocampus behind the optic chiasma were taken and embedded in paraffin. Continuous sections (4–5 µm) were cut and stained with hematoxylin and eosin (H&E). The morphology of neurons in CA1 and DG regions of the hippocampus and cerebral cortex was observed by using an optical microscope (Leica Microsystems Ltd., Wetzlar, Germany).

### 2.6 Nissl staining

The paraffin-embedded sections of the brain were deparaffinized and rehydrated in xylene and gradient alcohol solutions and then washed in PBS. The Nissl Stain Kit (Solarbio, Beijing, China) was used for Nissl staining. The Nissl bodies in the CA1 and DG regions of the hippocampus and cerebral cortex were examined under an optical microscope (Leica Microsystems Ltd., Wetzlar, Germany). For quantification, we used the ImageJ open-source software to analyze neurons.

### 2.7 RNA isolation and sequencing

TRIzol reagent (TaKaRa, Japan) was used to extract the total RNA of the hippocampus. NanoDrop, gel electrophoresis, and Agilent 2100 were used to ascertain 260/280 > 1.8; 260/230 > 1, RIN >7, and RNA >1 µg. Oligo dT beads and poly A were used to enrich mRNA. Fragmented mRNA (300 bp) samples were reverse-transcribed with random hexamers and reverse transcriptase to produce cDNA. The generated first-strand cDNA was co-reacted with RNase H enzyme, DNA polymerase I, and dNTPs to generate double-stranded cDNA. Purified double-stranded cDNA was subjected to end repair, the addition of a tail, and an adapter. The Illumina NovaSeq 6000 platform was used for RNA sequencing. Successful library construction was sequenced, and bioinformatics analyses were performed by Majorbio Biotech Co., Ltd. (Shanghai, China).

### 2.8 Principle component analysis

Principle component analysis (PCA) was performed with Partek Flow (Partek Inc., St. Louis, MO). The total raw gene counts from RNA sequencing (17700/sample) of 12 samples were imported into the Partek Flow server with SAMR1, SAMP8, SAMP8 + FMN-L (8 mg/kg), and SAMP8 + FMN-H (16 mg/kg) (three samples/group). Images of PCA were generated to visualize the differences in the gene expression pattern between groups (Kolli et al., 2021).

### 2.9 Differentially expressed gene analysis

RNA-seq data analysis was performed with the DESeq2 method compared to the SAMR1 group*.* The criteria for DEGs were set at Padj <0.05 and >1.2-fold.

### 2.10 Reverse transcription-quantitative polymerase chain reaction

Total RNA was quantified at 260/280 nm and reverse transcribed using the PrimerScript RT kit (TaKaRa Biotechnology Co., Ltd., Dalian, China). The primers were designed by Primer3 and synthesized by Sangon Biotech (Shanghai, China) (Supplementary Table S1). The PCR reaction system is as follows: 15 μL (7.5 μL SYBR super mix, 0.5 μL of 10 μM of each primer, 3 μL of cDNA, and 4 μL of DEPC water). The cycling conditions were one cycle of 95°C for 10 min; 95°C for 10 s, 60°C for 1 min for 40 cycles; 95°C for 1 min, 55°C for 1 min, and 55°C for 10 s for 80 cycles. The gene expression levels were determined by the quantification cycle values (Cq). The gene expression levels of glyceraldehyde-3-phosphate dehydrogenase (GAPDH) were used as the internal control. Each group was normalized to the SAMR1 group, which was set at 100%.

### 2.11 Ingenuity pathway analysis (IPA)

The ingenuity pathway analysis (IPA) server (QIAGEN, Redwood City, CA) was employed to perform canonical pathway and upstream regulator analyses. IPA calculates the significance using a right-tailed Fisher’s exact test. The *p*-value is the probability of the overlap between the treatment groups and the IPA pathway gene list. Moreover, upstream analysis used DEGs to predict upstream regulatory factors. The Z-score was used to evaluate changes among the treatment groups (Hattori et al., 2024).

### 2.12 BaseSpace correlation engine analyses

DEGs were uploaded to the BaseSpace Correlation Engine (BSCE) (https://www.illumina.com/products/by-type/informatics-products/basespace-correlationengine.html; formerly NextBio) and compared with all the biosets in the database using the running Fisher’s test (Kupershmidt et al., 2010). The curated study was filtered by organism (*Mus musculus*), data type (RNA expression), and keyword (Alzheimer’s disease). This method provides an assessment of the statistical significance of the correlation of the overlapping genes between each treatment’s DEGs and biosets curated in the Illumina Correlation Engine, with a summary *p*-value. The results were exported, and each *p*-value was converted to a –log (*p*-value). Biosets that were positively correlated with the DEGs were predicted to produce similar effects, either directly or indirectly; the larger the –log (*p*-value), the higher the degree of similarity. The top 10 positively correlated biosets were selected for heatmap visualization.

### 2.13 Statistical analysis

DEGs from RNA-seq were analyzed by DESeq2 as Padj ≤0.05 compared to SAMR1 (*p*-value after Benjamini & Hochberg correction). One-way analysis of variance (ANOVA) was used to determine the statistical differences among the groups, followed by Dunn’s multiple range tests. The data were expressed as mean ± SD. The significant criteria were set at *p* < 0.05.

## 3 Results

### 3.1 Effects of FMN on the learning and memory abilities and emotion in SAMP8 mice

**FIGURE 1:**
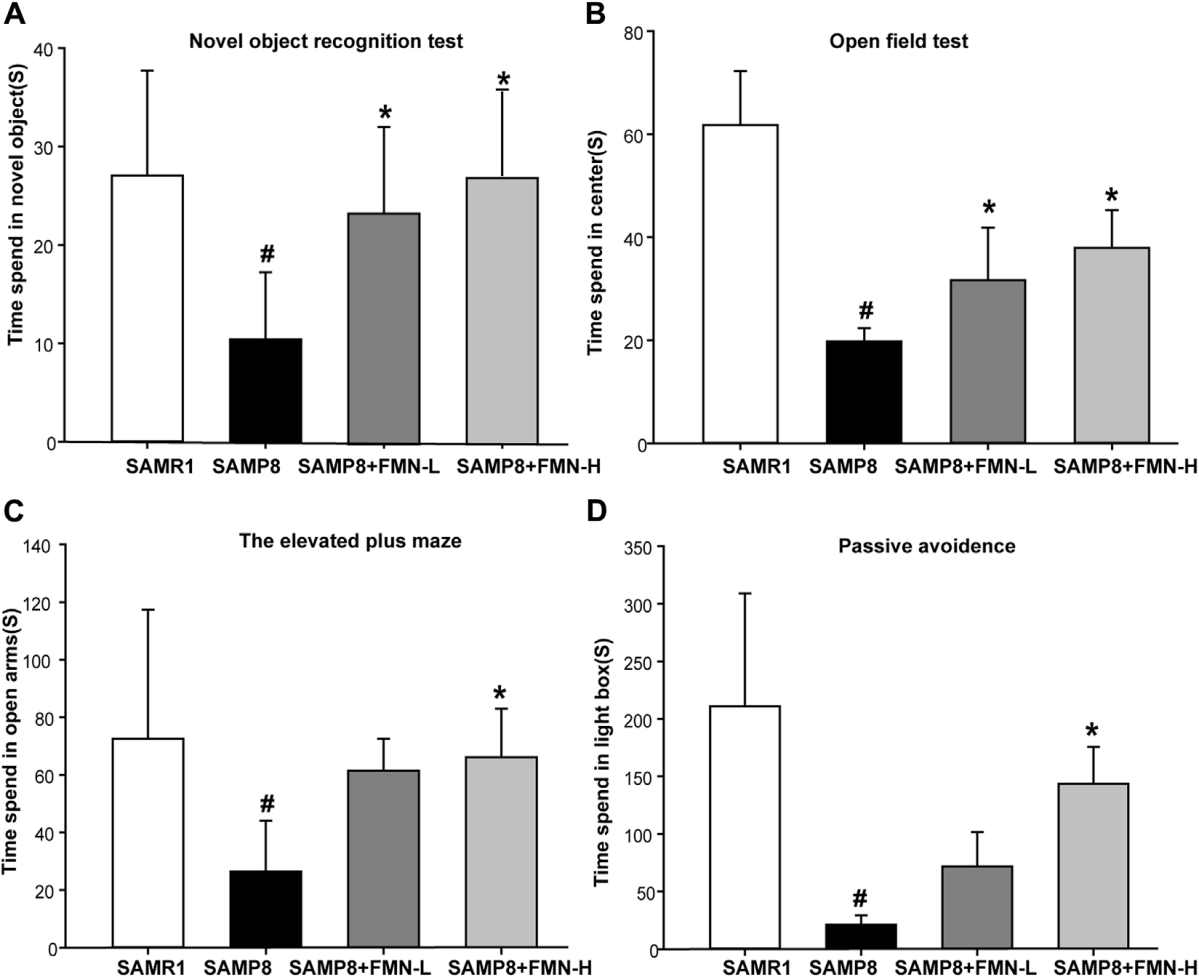
Effects of FMN on emotion and learning and memory in SAMP8 mice. **(A)** The activity time spent by mice in exploring the novel object. **(B)** The activity time spent by mice in the central region. **(C)** The activity time spent by mice in open arms. **(D)** The activity time spent by mice in the light box. Data are expressed as mean ± SD of n = 10, #*p* < 0.05 vs. SAMR1 mice, *p < 0.05 vs. SAMP8 mice.

The results of the NOR test revealed that compared with the SAMR1 mice, the learning and memory abilities of the 9-month-old SAMP8 mice decreased significantly (p < 0.05). The learning and memory abilities were obviously improved after 5 months of FMN treatment (8 and 16 mg/kg) (Figure 1A). The results of the OFT showed that the movement time of SAMP8 mice in the central region was significantly decreased compared with SAMR1 mice. After treatment with two concentrations of FMN, the reduction in movement time reduction for SAMP8 in the central region was reversed (Figure 1B).

The findings of the EPM test revealed that SAMP8 mice exhibited anxiolytic-like behaviors, as evidenced by a significant increase in the time spent in the open arms of the EPM compared with SAMR1 mice. Fortunately, 16 mg/kg FMN enhanced the time spent in the open arms (Figure 1C). PAT is another method to detect anxiety and depression in mice. The findings of PAT suggested that the time spent by mice in the light box was decreased significantly in the SAMP8 group. Moreover, FMN treatment increased the activity time of SAMP8 mice in the light box (Figure 1D). The above behavioral experiments showed that compared with SAMR1 mice, SAMP8 mice showed a loss of learning and memory abilities and changes in anxiety and depression. FMN could significantly improve the learning and memory abilities and ameliorate anxiety and depression in SAMP8 mice.

### 3.2 Effects of FMN on neuronal damage in SAMP8 mice

**FIGURE 2:**
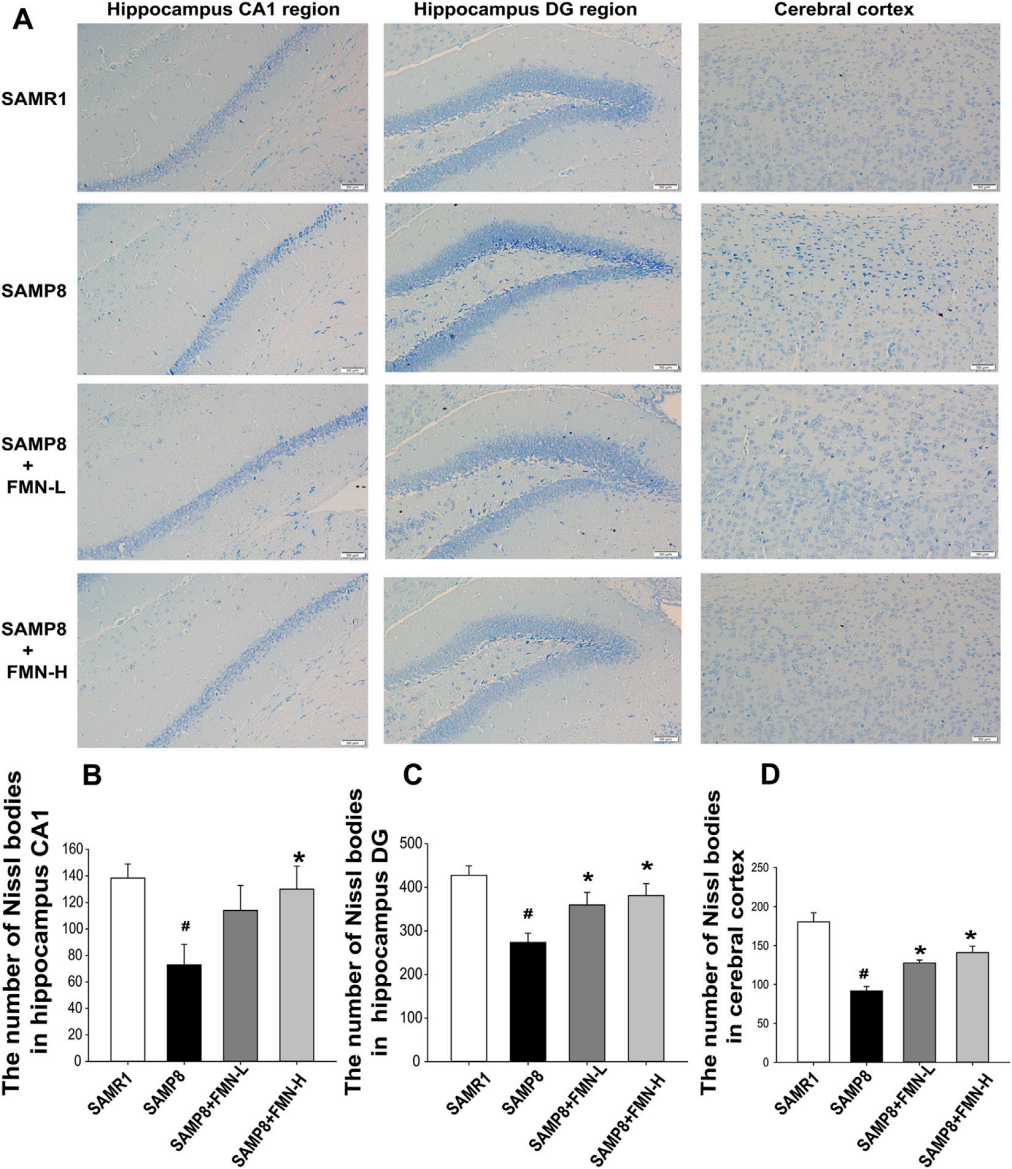
Effects of FMN on neuron morphology change in SAMP8 mice. **(A)** Representative images showing Nissl bodies in CA1 and DG regions of the hippocampus and cerebral cortex (magnification 200×, scale bar = 50 μm); **(B)** quantitation of Nissl-stained neurons in the normal form in the CA1 hippocampal region. **(C)** Representative images showing Nissl bodies in the DG hippocampal region. **(D)** Quantitation of Nissl-stained neurons in the normal form in the cerebral cortex. The numbers of Nissl bodies were captured in the three fields of the hippocampus CA1 and DG regions as well as in the cerebral cortex. ( $\bar{x}$ ± SD, n = 3), ^#^ *p* < 0.05 vs*.* SAMR1, ^*^ *p* < 0.05 vs*.* SAMP8 vehicle.

Neuronal morphology in CA1 and DG regions of the hippocampus and cerebral cortex were examined by Nissl staining. In the SAMR1 group, granule neurons exhibited round nuclei, which were located in the center of the perikaryon and surrounded by a pale cytoplasm. A significant loss of Nissl-stained neurons and vacuoles were observed in the 9-month-old SAMP8 mice. There were a large number of pyknotic neurons. Compared with SAMP8 mice, Nissl-stained neurons in the normal form in the CA1 and DG region of the hippocampus and cerebral cortex were increased after 4 months of treatment with both doses of FMN (8 and 16 mg/kg, Figure 2) (p < 0.05).

### 3.3 Principle component analysis

**FIGURE 3:**
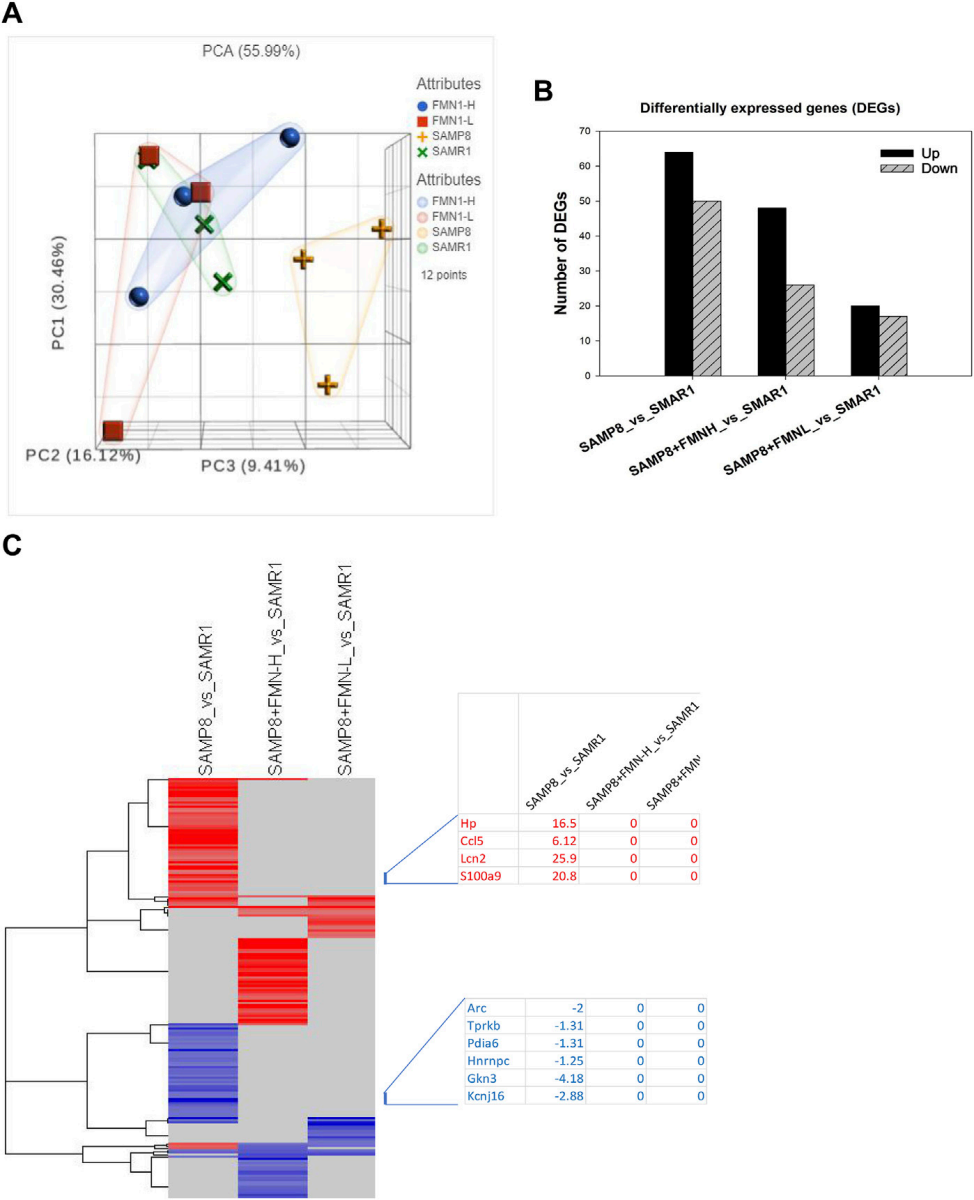
Differentially expressed genes. **(A)** Principle component analysis (PCA). **(B)** Differentially expressed genes (DEGs). **(C)** 2-D cluster heatmap of DEGs as compared to SAMR1 mice.

Brain RNA was extracted and subjected to RNA sequencing (Illumina HiSeq platform). FPKM (fragments per kilobase of exon per million fragments mapped) obtained from RNA-seq (17,674) was transformed into gene raw counts and clustered based on 12 individual samples and 4 groups. All gene counts were subjected to PCA. The PCA value is 55.99%, with PC1 = 30.46%, PC2 = 16.12%, and PC3 = 9.41%. The distributions of the SAMR1, SAMP8 + FMN-L (8 mg/kg), and SAMP8 + FMN-H (16 mg/kg) groups were apparently separated from the SAMP8 group (Figure 3A).

The DEGs were analyzed via the DESeq2 method compared to SAMR1. DEGs were screened using Padj ≤ 0.05. Compared to SAMR1, the SAMP8 group resulted in 114 DEGs (64 upregulated and 50 downregulated); SAMP8 + FMN-L (8 mg/kg) resulted in 37 DEGs (20 upregulated and 17 downregulated); and SAMP8 + FMN-H (16 mg/kg) resulted in 74 DEGs (48 upregulated and 26 downregulated) (Figure 3B).

**FIGURE 4:**
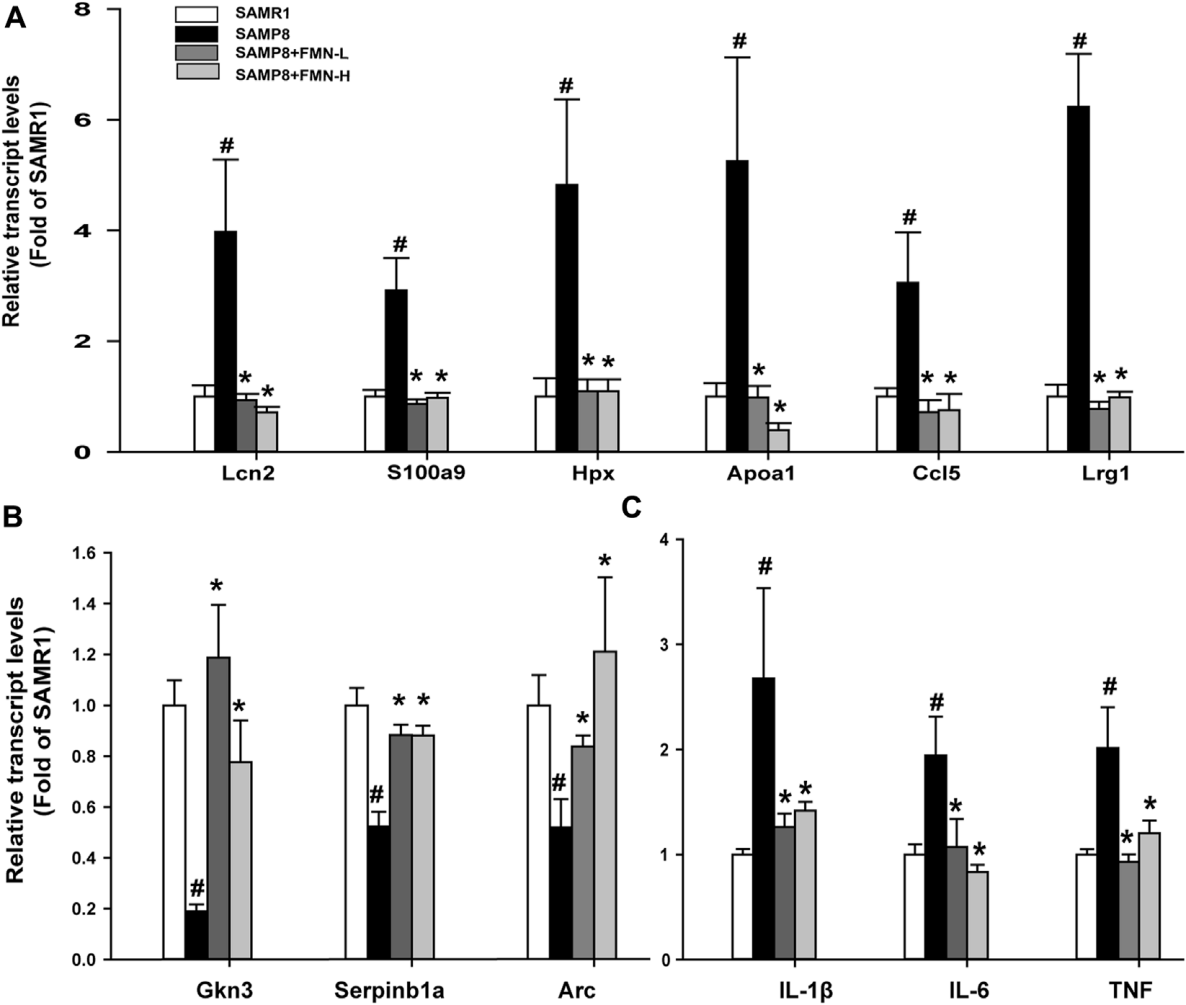
RT-qPCR analysis of 12 selected DEGs. **(A)** Upregulated genes in SAMP8 mice. **(B)** Downregulated genes. **(C)** Proinflammatory genes. Data are expressed as mean ± SD (n = 6). ^#^Significantly different from SAMR1, *p <* 0.05; *Significantly different from SAMP8, *p <* 0.05.

The 2-D clusters of DEGs are shown in Figure 4. It is obvious that FMN treatment attenuated aberrant gene expressions in SAMP8 mice. The first cluster is taken as an example; the increased gene expression in SAMP8 mice was abolished following FMN treatment, including overexpression of Lcn2 (25.9-fold), S100a9 (20.8-fold), Hp (16.5-fold), and Ccl5 (6.12-fold) and downregulation of Gkn3 (−4.18-fold), Kcnj16 (−2.88-fold), and Arc (−2-fold) (Figure 3C, left). The entire list of 197 DEGs given in Figure 4 can be found in Supplementary Table S2.

### 3.4 RT-qPCR analysis of selected DEGs

Based on the fold change (Supplementary Table S2), the selected 12 DEGs were further analyzed via RT-qPCR, as shown in Figure 4. Compared with the SAMR1 group, the expressions of Lcn2, S100a9, Hpx, Apoa1, Ccl5, and Lrg1 were increased in SAMP8 mice, and FMN treatments prevented such increases, regardless of dose levels (Figure 4A). The expressions of Gkn3, Serpinb1a, and Arc were decreased in the SAMP8 group, and both does of FMN reversed such decreases (Figure 4B). Moreover, the expressions of genes associated with inflammation (IL-1β, IL-6, and TNF) increased in the hippocampus of SAMP8 mice, which was attenuated by both doses of FMN (Figure 4C).

### 3.5 Ingenuity pathways analysis

**FIGURE 5:**
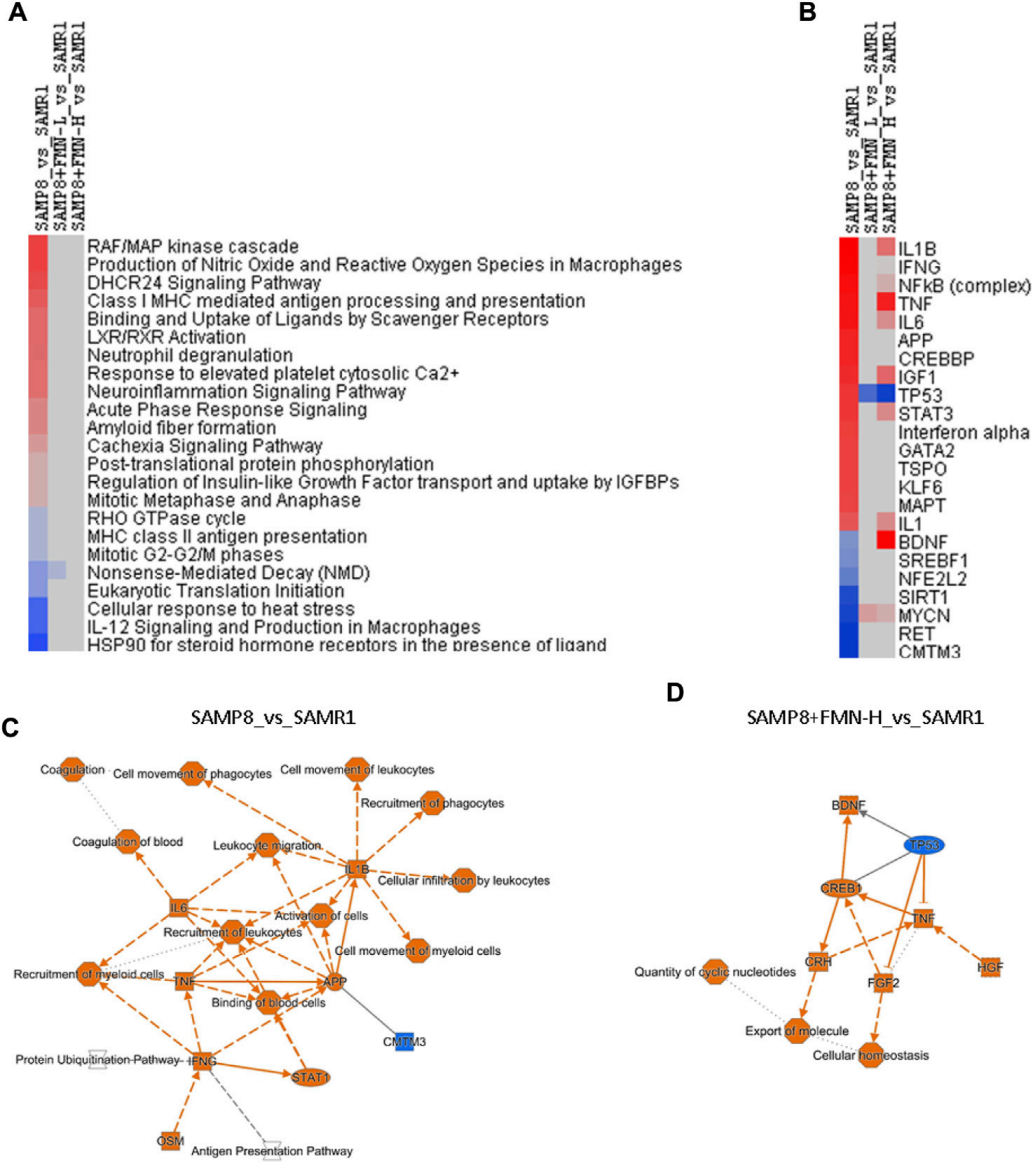
Ingenuity Pathway Analysis (IPA). **(A)** Selected canonical pathways. **(B)** Selected upstream regulators based on the Z-score. Red indicates upregulation, and blue indicates downregulation, and blank indicates no change, as compared to the SAMR1 group. **(C)** Graphical summary of SAMP8_vs._SAMR1 key gene interactions. **(D)** Graphical summary of SAMP8+FMN-H_vs._SAMR1 key gene interactions.

IPA is a web-based bioinformatics application that allows researchers to upload RNA-seq data for functional analysis, integration, and further understanding of the biological effects. Figure 5A illustrates the selected canonical pathways, including upregulated ones (red) in SAMP8 mice, such as “RAF/MAP kinase cascade,” “production of nitric oxide and reactive oxygen species in macrophages,” “DHCR24 signaling pathway,” “LXR/RXR activation,” “neuroinflammation signaling pathway,” “acute phase response signaling,” and “amyloid fiber formation.” Downregulated pathways in SAMP8 mice (blue) include “HSP90 for steroid hormone receptors,” “IL-12 signaling in macrophages,” and “cellular responses at stress”. All the abnormal pathways were attenuated or brought to normal after FMN treatment (gray in color).

Figure 5B illustrates the selected upstream regulators that may be responsible for gene expression changes observed in the study. Compared with SAMR1, increases in SAMP8 mice include “IL1B,” “IFNG,” “NFκB complex,” “TNF,” “IL6,” “APP,” “STAT3,” “MAPT,” and “IL-1.” Decreases in SAMP8 mice include “CMTM3,” “RET,” “MYCN,” “SIRT1,” NFE2L2,” “SREBF1,” and “BDNF.” Both doses of FMN attenuated the abnormalities (light color), brought to normal (gray), and even changed “TP53,” “BDNF,” and “MYCN” to the opposite color.

Figure 5C shows a graphic summary of gene interactions between SAMP8 and SAMR1 mice. The activation of IL-1B, IL-6, TNF, and APP and downregulation of CMTM3 were the main molecular events, implying neuroinflammation, immune disruption, and β-amyloid pathogenesis. After being treated with FMN, the activation of BDNF, CREB1, HGF, FGF2, and CRH and downregulation of TP53 were the major events against neuroinflammation, promoting neurogenesis and repair (Figure 5D).

### 3.6 Correlation with the GEO database

**FIGURE 6:**
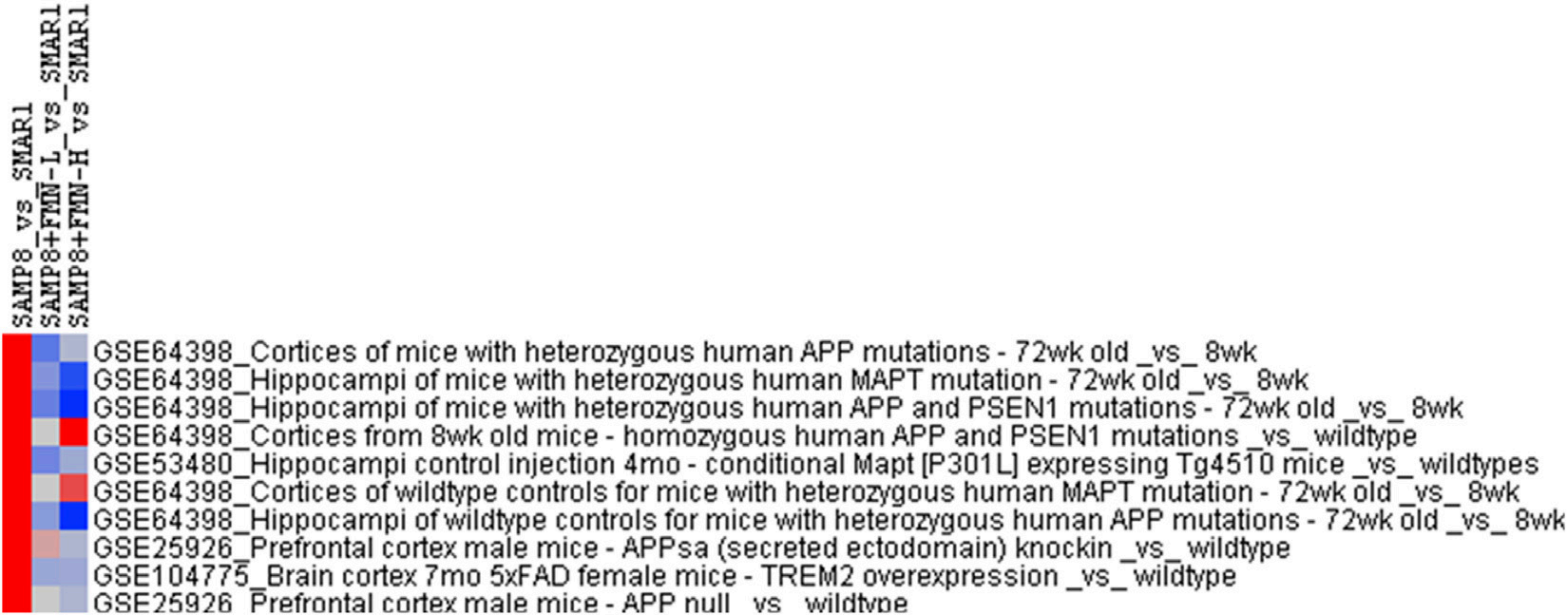
Correlation with the GEO database analysis. The DEGs were imported into the Illumina Correlation Engine to compare the gene expression biosets in the GEO database using –log (*p*-value), with upregulation shown in red and downregulation shown in blue. The correlations were in the format of the GSE number. Study name in the GEO database.

The DEGs were imported into the Illumina BaseSpace Correlation Engine (BSCE) for curated studies with the GEO database and filtered by “*M. musculus*,” “RNA expression,” and “Alzheimer’s disease.” –Log (p-values) values were calculated for comparison. SAMP8_vs._SAMR1 had 48 curated studies with *p*-values >4 or <4, and the top 10 biosets are illustrated in Figure 6. The deeper the red color, the higher the –log (*p*-values), and the deeper blue color indicates a lower –log (p-values).

The DEGs of SAMP8_vs_SAMR1 were highly correlated with GSE64398 (Matarin et al., 2015), in that –log (p-values) from cortices of APP mutant mice (11.0), APP and PSEN1 mutants (6.69) MAPT mutant (6.14), from hippocampi of MAPT mutant (8.23), APP and PSEN1 mutants (7.28), and APP mutant (5.92) were abolished or attenuated by FMN treatment. GSE53480 (Polito et al., 2014) had a –log (*p*-value) of 6.70 from hippocampi of MAPT expression Tg4510 mice, and GSE25926 (Aydin et al., 2011) had a –log (*p*-value) of 5.72 from the prefrontal cortex of MAPT expression Tg4510 mice and 4.64 from the prefrontal cortex of APP mice. GSE104775 (Lee et al., 2018) had a –log (*p*-value) of 5.37 from the cortex of 7 mo 5 × FAD mice with TREM2 overexpression. FMN treatment at both doses attenuated such correlations, even with negative values, supporting the beneficial effects of FMN.

## 4 Discussion

The present study verified cognition deficits and neuron loss in 9-month-old SAMP8 mice compared to age-matched SAMR1 mice. FMN treatments of SAMP8 mice alleviated behavioral deficits and prevented neuronal loss in the cortex and hippocampal CA1 and DG regions. Gene expression in SAMP8 was quite different from that of SAMR1 mice with 114 DEGs under Padj ≤0.05. FMN treatments attenuated or abolished these changes. The qPCR of selected genes verified RNA-seq. IPA revealed activation of IL-1B, IL-6, TNF, and APP and downregulation of CMTM3, Nrf2, and Sirt-1 signaling in SAMP8 mice, while FMN activated CREB1–BDNF signaling growth factors along with the downregulation of TP53 to combat AD-like pathology. The gene expression profile of SAMP8 mice was correlated with that of other AD mouse models in the GEO database. Thus, this study revealed major molecular changes associated with cognitive deficits in aged SAMP8 mice and clearly demonstrated FMN protection by targeting these abnormalities.

The cognition impairments of the SAMP8 model developed with aging. No apparent cognitive deficit was evident in young (4-month-old) SAMP8 mice compared to 9-month-old SAMP8 mice (Yanai and Endo, 2016; Liu et al., 2020b). The long-term intervention strategy in our prior studies (Liu et al., 2020a; Liu et al., 2020b; Lv et al., 2020; Liu et al., 2023) was therefore used. This study clearly showed that FMN was effective in preserving the learning and memory abilities and ameliorating anxiety and depression in SAMP8 mice, which is consistent with the role of FMN in improving cognitive deficits in APP/PS1 mice (Fei et al., 2018). Hippocampal CA1 interneuron’s role in local circuit computation is important for spatial learning behavior (Jeong and Singer, 2022), and the hippocampal DG region plays a key role in memory function (Hainmueller and Bartos, 2020). Meanwhile, hippocampal abnormalities have been implicated in anxiety and depression (Tang et al., 2019). The cerebral cortex is also responsible for processing motor and sensory information that supports high-level cognitive abilities and shapes personality (Stoufflet et al., 2023). Therefore, we performed a histopathological examination on the CA1 and DG region of the hippocampus and cerebral cortex. The results of H&E and Nissl staining further confirmed the protective effects of FMN on neurons in the hippocampus CA1 and DG regions and cerebral cortex of SAMP8 mice. The behavioral tests and histopathological examination paved the road for further RNA-seq analysis and result interpretation. It is well known that the hippocampus is a key area of episodic memory formation and the site for learning and memory (Lazarov et al., 2024), and the whole hippocampus was used for transcriptome analysis.

The 2-D gene cluster of DEGs shows that genes associated with immunity and acute phase response (e.g., Lcn2, S100a9, Ccl5, and Lrg1) and metabolism (e.g., Hpx and Apoa1) were increased, but other adaptive response gene expressions (e.g., Gkn3, Serpinb1a, and Arc) were decreased in SAMP8 mice. Both doses of FMN restored the abnormal gene expressions of SAMP8 mice to the normal level. RT-qPCR was used to verify 12 selected genes, confirming the findings of RNA-seq . For example, Lcn2 (lipocalin 2), an innate immune protein, plays a pivotal role in promoting sterile inflammation by regulating immune responses (Xu et al., 2020) and contributes to the onset and progression of AD in the amyloid-beta oligomer-induced mouse model (Kang et al., 2021). S100a9 is mainly produced by innate immune cells, which possess intrinsic amyloidogenic properties and the ability to modulate Aβ aggregation and can serve as a prospective therapeutic target for AD (Wang et al., 2014). CCL5, an important chemokine for the modulation of inflammatory responses, plays an important role in normal physiologic processes as well as pathologic conditions in the pathogenesis of AD (Ma et al., 2023). Apolipoprotein A-1 (Apoa1) and hemopexin (Hpx) are associated with cholesterol metabolism, and both are considered cerebrospinal fluid markers for AD (Roher et al., 2009). The results of RT-qPCR confirmed that the genes related to immunity and metabolism in SAMP8 mice were abnormally expressed compared to SAMR1 mice, but the abnormal gene expressions were ameliorated after FMN. On the other hand, FMN enhances synaptic plasticity and resistance to inflammation by activating the expressions of adaptive response genes such as Gkn3 (Gastrokine 3) (Mukherjee et al., 2018), Serpinb1a (Zhao et al., 2014), and Arc that mediate synapse-specific signaling and metaplasticity contributing to memory updating and disrupted in cognitive disease (Yang et al., 2023). Proinflammatory cytokines IL-1β, IL-6, and TNF were increased in SAMP8 compared to SAMR1 mice, which is consistent with the literature findings (Tha et al., 2000), but FMN treatment abolished such increases.

IPA revealed the alteration of canonical pathways and upstream regulators in SAMP8 mice and pointed toward neuroinflammation and amyloid pathology, as well as compromised adaptive responses and SIRT-1 signaling in aged SAMP8 mice (Figures 5A, B). A graphic summary of IPA could help us understand molecular events in SAMP8 mice (Figure 5C). All proinflammatory mediators and AD-related APP were increased (orange in color), which is consistent with the activation of IL-1β, IL6, TNF, IFNG, STAT1, and OSM (oncostatin) in aged SAMP8 mice (Tha et al., 2000; Liu et al., 2017; Jiang et al., 2018). Behavioral deficits in SAMP8 mice are associated with the overexpression of APP and BACE1 (Ha et al., 2020). The only downregulation in SAMP8 mice was of CMTM3 (blue in color), which acts as a new immune checkpoint regulator (Shen et al., 2022), suggesting the disruption of immune homeostasis.

A concise graphic summary generated by IPA could help us understand FMN effects (Figure 5D). After FMN treatment, the expressions of CREB1 and BDNF increased. CREB and BDNF play important roles in neurobiology, and targeting CREB–BDNF signaling could be a promising treatment of AD (Amidfar et al., 2020). Other upregulated molecules include HGF, FGF2, and CRH. Reduced HGF/MET signaling contributes to synaptic pathology in the 5 × FAD mouse model of AD (Wei et al., 2022); while FGF2 gene transfer restores hippocampal functions in APP/PS1 mice (Kiyota et al., 2011); CRH (corticotropin-releasing hormone) has been proposed to play important roles in AD and other dementias (Rehman, 2002). FMN upregulation of these key molecules could be the mechanism of protection. Increased TNF could be a double-edged sword in FMN’s effects. Dysregulation of TP53 plays an important role in AD pathogenesis (Abate et al., 2020). In upstream analysis (Figure 5B), TP53 was increased, while SIRT1 and NRF2 were decreased in aged SAMP8 mice, and FMN reversed these abnormalities, which is consistent with the distribution of NRF2 through the SIRT1–P53 pathway in aged SAMP8 mice (Wang et al., 2024),which could be targets for FMN.

The Illumina BaseSpace Correlation Engine (BSCE) (https://www.illumina.com/products/by-type/informatics-products/basespace-correlationengi ne.html; formerly NextBio) curates over 23,000 scientific studies of RNA sequencing and microarray database to provide data-driven correlations for genes, experiments, drugs, and phenotypes for the research (Corton et al., 2019). In the present study, the differential gene expression profiles of the hippocampus of SAMP8 mice were highly correlated with those of the hippocampus or cortex of other AD mouse models, including APP mutant mice, APP and PSEN1 mutant mice, and MATP mutant mice (Matarin et al., 2015); the Tg4510 mouse model of tauopathy (Polito et al., 2014); APP family member mutant mice (Aydin et al., 2011), and aged 5 × FAD mice (Lee et al., 2018), implying the similarities of the SAMP8 mouse model with various AD mouse models in molecular mechanisms, Nonetheless, FMN treatment at both doses attenuated such correlations, even with negative values, further supporting the beneficial effects of FMN. Although SAMP8 mice is a good model for AD drug research (Liu et al., 2020b), it may not be fully representative of the complexity of human AD, and clinical studies of FMN’s protective effects warrant further investigation.

In conclusion, the present RNA-seq study revealed major molecular events associated with cognitive deficits in aged SAMP8 mice, which could be targets of therapeutic candidates like FMN. FMN in combination with other compounds could provide a better therapeutic strategy for AD.

## Data Availability

The datasets presented in this study can be found in online repositories. The names of the repository/repositories and accession number(s) can be found in the article/Supplementary Material.
