# Supplementary material for: Transcriptome analysis of the aged SAMP8 mouse model of Alzheimer’s disease reveals novel molecular targets of formononetin protection
Source: Front Pharmacol. 2024 Aug 21;15:1440515. doi: 10.3389/fphar.2024.1440515 (PMC11371586; doi:10.3389/fphar.2024.1440515)
Supplement: Supplementary file 1 [file DataSheet1.docx]

**Supl Fig.1. Effects of FMN on** **neuronal damage in the brain of SAMP8 mice**

As indicated in Supl Fig. 1, compared with SAMR1 mice, loose structures neurons with disordered pyramidal cell layers occurred in CA1 and DG regions of hippocampus and cerebral cortex of SAMP8 mice. Furthermore, the number of neurons were reduced, accompanied by nuclear condensation in SAMP8 mice. FMN treatment group significantly alleviated the pathological damage described above.


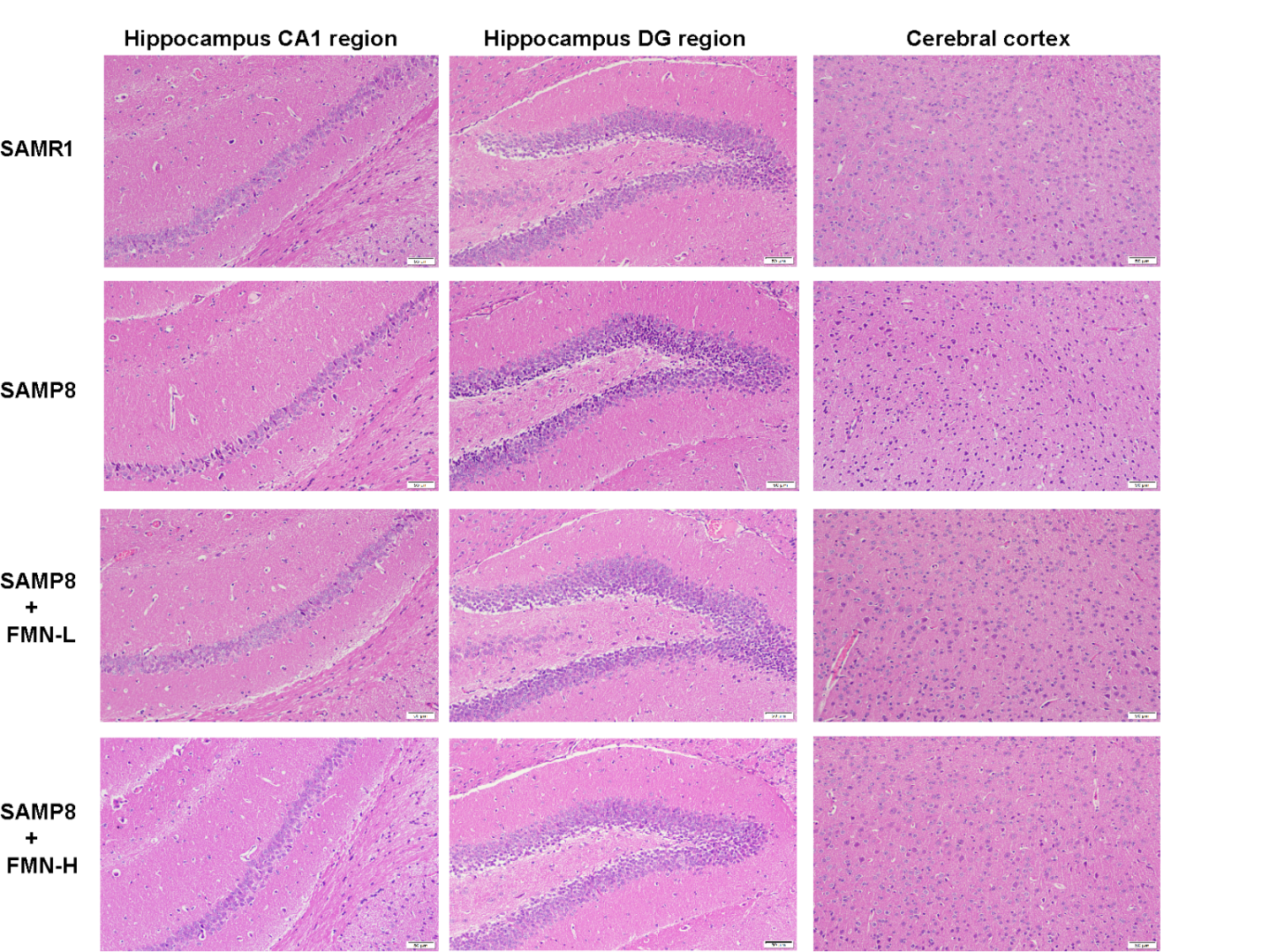


**Supl Fig.1. The effects of FMN on** **neuronal damage of the** **CA1 and DG regions of hippocampus and cerebral cortex of SAMP8 mice.** Mice were orally pretreated with FMN (8 mg/kg) and FMN (16 mg/kg) for 5 months. The brain tissues were stained with hematoxylin and eosin, and examined under light microscope. Magnitude (200×).

Supplementary Table 2 2D-cluster of differentially expressed genes (First cluster, all 197 in X-cell file)
